# Short-timescale heart-rate fluctuation and acoustic stability show structured coupling at register transitions during singing

**DOI:** 10.3389/fpsyg.2026.1783731

**Published:** 2026-08-13

**Authors:** Youhe Li

**Affiliations:** Shenzhen Technology University, Shenzhen, China

**Keywords:** acoustic analysis, embodied performance, heart rate, multimodal analysis, performance science, physiological arousal, singing, vocal register

## Abstract

Heart rate (HR) is widely used to index physiological activation during singing and music performance, yet condition-level mean HR provides limited insight into how physiological regulation unfolds during vocal task execution. This exploratory study examined whether short-timescale changes in HR and acoustic stability form a structured coupling pattern at vocal register transitions, and whether that structure is shared across singers or varies across individuals and task contexts. Twenty-five trained singers completed structured vocal tasks under low-, moderate-, and high-arousal conditions. Analyses focused on Low→ Middle and Middle→ High register-transition events. Short-timescale HR change was represented by the source-to-target difference in signed maximal short-timescale HR slope (Δ*s*), whereas acoustic change was represented by the source-to-target difference in the composite acoustic-stability score (Δ*A*). Across 150 transition events, 72 were classified as aligned, 77 as opposed, and 1 as a tie. The aligned-minus-opposed proportion difference was −0.034, with a participant-cluster bootstrap 95% confidence interval of [−0.230, 0.160], providing no evidence for one dominant coupling direction. At the same time, the continuous association between Δ*s* and Δ*A* was weakly negative, Pearson's *r* = −0.180, with a participant-cluster bootstrap 95% confidence interval of [−0.317, −0.040]. Individual profiles were heterogeneous, with more than half of the singers showing mixed rather than consistently aligned or opposed coupling. These findings indicate that short-timescale HR fluctuation and acoustic stability are modestly but observably structured at register transitions, without supporting a single dominant group-level coupling direction. Physiological–acoustic coupling in singing is therefore better understood as task- and person-sensitive rather than as a uniform response or fixed physiological target. This event-based framework provides an empirical basis for person-specific regulatory profiling, multimodal performance analysis, and future human-in-the-loop computational approaches to vocal assessment and feedback.

## 1 Introduction

Singing is an embodied sensorimotor activity that requires the coordination of vocal production with auditory and somatosensory feedback (Blanco et al., 2021; Sundberg, 1987; Zarate and Zatorre, 2008). Respiratory behavior and vocal production may also vary across singers and task contexts (Traser et al., 2022). Register transitions are especially informative moments within this process because register-related contrasts cannot be reduced to movement between pitch levels alone. Differences among vocal registers can persist across overlapping ranges of fundamental frequency and involve broader changes in vocal production and acoustic configuration (Lee et al., 2023; Lehoux et al., 2024; Titze, 2000). The demands associated with a register-transition task may additionally vary with training history, task familiarity, and performance context (Crocco et al., 2020; Zuim et al., 2024). A transition that is habitual and relatively stable for one singer may therefore require greater adjustment from another. Register transitions provide a structured but person-sensitive context in which short-timescale physiological regulation and acoustic output can be examined together.

Physiological arousal is one component of this performance context. In the present study, it was operationalized as experimentally induced short-term variation in physiological activation across low-, moderate-, and high-arousal task blocks and was evaluated at the block level using participant-level mean singing heart rate (HR). These blocks were not assumed to impose an identical physiological dose on every singer; rather, they were designed to create distinguishable task contexts while preserving the capacity for controlled phonation.

Music-related cardiovascular responses have been examined across listening, audience, instrumental-performance, and vocal-production contexts (Bernardi et al., 2006, 2017; Iñesta et al., 2008; Kulinski et al., 2022; Shoda et al., 2016). These studies indicate that HR responses can vary with musical setting, task demand, performance context, and individual characteristics, although their populations and measurement conditions differ. Multimodal performance studies have further combined physiological information with musical, performance-related, or qualitative evidence (Horwitz et al., 2021; Soliński et al., 2024). Together, this literature supports examining HR as one component of performance-related physiological activation while retaining clear boundaries around task context and measurement resolution.

Mean HR is useful for characterizing general activation at the task or block level, but it provides limited information about how physiological regulation changes during task execution (Achten and Jeukendrup, 2003). Singing requires recurrent adjustments in breathing, phonation, pitch control, and register coordination, and these adjustments may occur over shorter intervals than block-level averages can represent (Sundberg, 1987; Titze, 2000). Two singers may therefore show similar mean HR while exhibiting different short-term patterns of physiological adjustment. Music-related physiological responses can also vary across individuals and performance contexts (Iñesta et al., 2008; Nomura, 2024). Examining short-timescale HR dynamics may therefore complement mean HR by describing how the dominant direction and magnitude of HR fluctuation change across successive task segments.

The acoustic side of this relationship also requires a bounded interpretation. Acoustic stability can be operationalized through measurable features such as pitch variability, jitter, shimmer, and harmonics-to-noise ratio, but isolated acoustic measures do not constitute a comprehensive evaluation of perceived vocal quality, expressive intention, or technical success, all of which depend on broader perceptual and contextual factors (Bruder et al., 2024; Himonides, 2019). Their value in the present study lies in describing relative within-participant changes in voice consistency across register segments. The central question is therefore not whether a particular HR direction is inherently beneficial or whether a higher acoustic score represents universally better singing. Rather, it is whether transition-level changes in short-timescale HR fluctuation and acoustic stability show an observable coupling structure, and whether that structure is consistent or heterogeneous across singers and task contexts.

Accordingly, the present exploratory study examined 25 trained singers performing structured vocal tasks under low-, moderate-, and high-arousal task blocks. The analysis focused on Low→ Middle and Middle→ High register-transition events and examined transition-level changes in signed maximal short-timescale HR slope (Δ*s*) with changes in acoustic stability (Δ*A*). We examined the continuous association between Δ*s* and Δ*A*, the distribution of aligned and opposed coupling patterns, and the extent to which individual singers showed dominant or mixed coupling profiles. The study did not assume one optimal physiological direction or seek to infer a specific autonomic mechanism. Instead, it aimed to determine whether physiological–acoustic coupling at register transitions showed an observable structure, how strongly that structure was expressed, and how it varied across individuals and task contexts.

## 2 Methods

### 2.1 Participants

Twenty-five trained adult singers aged 18–21 years participated in the study. The sample included 19 women and 6 men, all of whom were undergraduate vocal-performance majors specializing in either Western classical singing or Chinese national singing. Voice types comprised 18 sopranos, 1 mezzo-soprano, 3 tenors, and 3 baritones. All participants reported normal hearing and no history of diagnosed voice disorders or cardiovascular, respiratory, or neurological conditions.

Before participation, singers were instructed to obtain adequate rest, refrain from caffeine, strong tea, alcohol, and energy drinks for at least 24 h, avoid strenuous exercise, and wear comfortable, loose-fitting clothing suitable for the activity-based induction tasks. All participants provided written informed consent. The study was conducted in accordance with the Declaration of Helsinki and local institutional requirements.

Recruitment targeted trained undergraduate vocal-performance students in order to maintain a broadly comparable level of training background and task familiarity. Individual differences in voice type, vocal technique, register-control difficulty, and physiological response were nevertheless expected to remain. The within-participant design reduced the influence of between-participant baseline differences, and participant-level clustering was preserved in the analyses.

### 2.2 Experimental design and procedure

**Figure 1:**
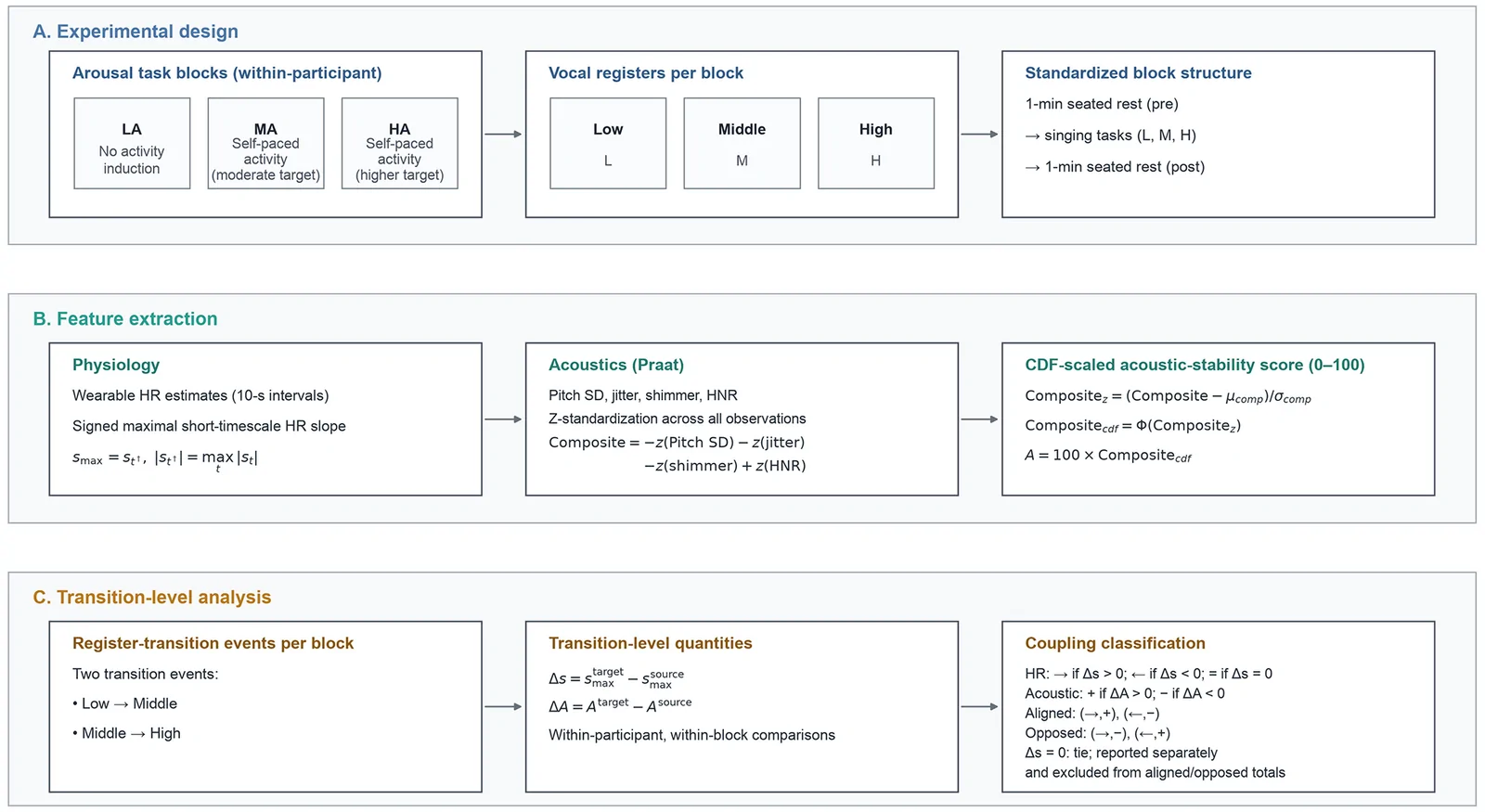
*Experimental design and analysis pipeline*. Participants completed three fixed-order arousal task blocks (LA, MA, and HA), each containing Low-, Middle-, and High-register productions. The blocks represented structured task contexts and were not assumed to impose identical physiological doses across participants. HR was represented by 10-s interval-averaged estimates, from which the signed maximal short-timescale HR slope was calculated for each register segment. Acoustic stability was summarized as a 0–100 CDF-scaled acoustic-stability score **(A)** derived from Pitch SD, jitter, shimmer, and HNR standardized across all observations. For each block, Δ*s* and Δ*A* were calculated for the Low→ Middle and Middle→ High transitions. Non-tie events were grouped as aligned or opposed, whereas events with Δ*s* = 0 were reported separately as ties. LA, low arousal; MA, moderate arousal; HA, high arousal; HR, heart rate; Pitch SD, pitch standard deviation; CDF, cumulative distribution function; HNR, harmonics-to-noise ratio.

A within-participant design was used, with three arousal conditions—low arousal (LA), moderate arousal (MA), and high arousal (HA)—crossed with three task-defined vocal registers: Low, Middle, and High. Each participant completed the vocal task in all nine arousal-by-register combinations. The arousal blocks were administered in a fixed LA → MA → HA order to reduce the possibility that residual activation from a higher-arousal block would carry over into a subsequent lower-arousal block. An overview of the experimental design and analysis pipeline is provided in Figure 1.

The timing structure and task sequence were standardized across participants. Each block began with a 1-min seated baseline period and ended with a 1-min seated recovery period after singing. In the LA block, participants proceeded directly from the seated baseline to the vocal task. In the MA and HA blocks, the baseline period was followed by 5 min of whole-body activity, such as brisk stepping, jogging in place, or comparable calisthenic movements. Participants then completed a 1-min seated interval before beginning the vocal task.

Exercise intensity was individually adjusted within protocol-defined HR target ranges. Participant-specific implementation was informed by the pre-session questionnaire, physical capacity, and real-time HR response, while the target ranges remained those specified in the study protocol. The ranges were informed by the commonly used age-predicted maximal-HR heuristic of 220 minus age and by general exercise-prescription guidance (Garber et al., 2011; Martin et al., 2025). Operational targets were below 100 bpm for LA, 100–115 bpm for MA, and 115–140 bpm or higher for HA. These values were used to guide implementation rather than as rigid physiological thresholds.

Participants were instructed to perform the activity as needed but not to exhaustion. The HA block was treated as the intended higher-arousal task block rather than as a requirement that every participant exceed 140 bpm. Throughout the induction procedure, experimenters monitored whether participants remained able to complete the subsequent vocal task with controlled phonation. Participant-level mean singing HR across the three blocks was subsequently evaluated as an arousal-manipulation check (Supplementary Table S2; Supplementary Figure S2).

Within each arousal block, participants completed the same sequence of Low-, Middle-, and High-register productions. The individually selected vowel and register-specific pitches were held constant across the LA, MA, and HA blocks, and the same duration and recording-quality requirements were applied in every block. Detailed vocal-task and recording procedures are described below.

### 2.3 Vocal tasks and recording

Within each arousal block, participants produced sustained vowels in three task-defined vocal registers: Low, Middle, and High. Each participant selected one comfortable vowel for the vocal task and one representative pitch for each register. The register-specific pitches were selected within the protocol-defined ranges appropriate to the participant's voice type. Once selected, the vowel and pitches remained unchanged across the LA, MA, and HA blocks, allowing within-participant comparisons under consistent vocal-task conditions. The experimental tasks consisted of controlled sustained-vowel exercises rather than performances of complete songs or specific repertoire. Western classical singing and Chinese national singing referred to the participants' vocal-training specializations rather than to the musical materials used in the experiment. Lyrics, musical interpretation, and expressive direction were not manipulated as experimental variables.

For each register, participants produced two short sustained vowels (1.5 s) and two long sustained vowels (5 s), all of which were retained as valid samples. Participants repeated the task when necessary until valid recordings meeting the predefined acceptability criteria were obtained. More than the minimum number of trials was typically recorded so that valid segments and backup samples were available.

Low, Middle, and High were operationalized as task-defined vocal settings within each singer's own voice type. The selected pitches served as within-participant task anchors and did not represent identical absolute pitch regions across voice categories. They were not intended to define vocal register solely by pitch frequency. Register-related contrasts can persist across equivalent or overlapping fundamental-frequency ranges and involve differences in vocal production and acoustic configuration (Lee et al., 2023; Lehoux et al., 2024; Titze, 2000). Low, Middle, and High were therefore treated as singer-specific vocal contexts rather than as pitch categories alone. Register-specific task pitches and their sounding frequencies are reported in Table 1.

**Table 1:** Register-specific task pitches and sounding frequencies by participant.

ID	Voice type	Low register	Middle register	High register
01	Soprano	C4, 261.63 Hz	A4, 440.00 Hz	E5, 659.26 Hz
02	Soprano	D4, 293.66 Hz	G4, 392.00 Hz	E5, 659.26 Hz
03	Tenor	C3, 130.81 Hz	A3, 220.00 Hz	E4, 329.63 Hz
04	Baritone	A2, 110.00 Hz	F3, 174.61 Hz	E4, 329.63 Hz
05	Baritone	A2, 110.00 Hz	G3, 196.00 Hz	D4, 293.66 Hz
06	Soprano	E4, 329.63 Hz	D5, 587.33 Hz	F5, 698.46 Hz
07	Soprano	C4, 261.63 Hz	A4, 440.00 Hz	E5, 659.26 Hz
08	Soprano	E4, 329.63 Hz	A4, 440.00 Hz	E5, 659.26 Hz
09	Soprano	C4, 261.63 Hz	G4, 392.00 Hz	E5, 659.26 Hz
10	Soprano	E4, 329.63 Hz	A4, 440.00 Hz	E5, 659.26 Hz
11	Soprano	A3, 220.00 Hz	F4, 349.23 Hz	E5, 659.26 Hz
12	Soprano	E4, 329.63 Hz	F4, 349.23 Hz	E5, 659.26 Hz
13	Tenor	D3, 146.83 Hz	C4, 261.63 Hz	G4, 392.00 Hz
14	Tenor	B2, 123.47 Hz	C4, 261.63 Hz	G4, 392.00 Hz
15	Soprano	C4, 261.63 Hz	F4, 349.23 Hz	E5, 659.26 Hz
16	Soprano	D4, 293.66 Hz	C5, 523.25 Hz	E5, 659.26 Hz
17	Soprano	C4, 261.63 Hz	A4, 440.00 Hz	E5, 659.26 Hz
18	Soprano	A3, 220.00 Hz	A4, 440.00 Hz	E5, 659.26 Hz
19	Soprano	D4, 293.66 Hz	G4, 392.00 Hz	E5, 659.26 Hz
20	Soprano	C4, 261.63 Hz	A4, 440.00 Hz	E5, 659.26 Hz
21	Soprano	D4, 293.66 Hz	C5, 523.25 Hz	E5, 659.26 Hz
22	Soprano	B3, 246.94 Hz	G4, 392.00 Hz	E5, 659.26 Hz
23	Mezzo-soprano	E4, 329.63 Hz	C5, 523.25 Hz	D5, 587.33 Hz
24	Baritone	G2, 98.00 Hz	G3, 196.00 Hz	E4, 329.63 Hz
25	Soprano	E4, 329.63 Hz	C5, 523.25 Hz	E5, 659.26 Hz

ID, participant identifier; Hz, hertz. Pitches are reported using sounding scientific pitch notation with corresponding frequencies calculated in 12-tone equal temperament using A4 = 440 Hz. The frequencies are provided as task anchors for transparency and should not be interpreted as defining vocal register solely by pitch height.

Recordings were made in an acoustically treated studio using a Shure MV7 microphone at a sampling rate of 48 kHz and a bit depth of 24 bits. The microphone was positioned 20 cm from the participant's mouth at a fixed angle. Microphone placement, gain, and room conditions were kept constant across participants and arousal blocks.

### 2.4 Heart rate recording and preprocessing

Heart rate was recorded using a first-generation Apple Watch Ultra worn on the non-dominant wrist with consistent strap tension. The watch's optical heart-rate function is based on wrist photoplethysmography (PPG), which estimates pulse-related changes in peripheral blood volume rather than cardiac electrical activity measured by electrocardiography (ECG; Charlton et al., 2022). HR values were exported with timestamps and represented interval-averaged estimates at 10-s time steps. The HR series were aligned with the experimental timeline so that samples corresponding to the predefined singing periods could be identified.

The HR data were screened for missing values and abrupt discontinuities inconsistent with adjacent observations and the recorded session timeline. Only observations falling within the predefined singing windows, from the task-start cue to the end of the relevant singing segment, were used to derive short-timescale HR measures.

When an isolated missing value prevented calculation from an immediately adjacent 10-s pair, the nearest valid pair was used. If the valid observations were separated by 20 s, the HR difference was divided by 2 to express the change as a 10-s equivalent. The specific pair used in such cases was documented during data annotation.

Agreement between wrist-PPG HR estimates and ECG reference measurements can vary with movement, physical-activity context, and measurement window (Alfonso et al., 2022; Lambe et al., 2026). The present analyses were therefore restricted to descriptive changes derived from the exported 10-s HR series and did not treat the measurements as ECG-equivalent or beat-to-beat recordings. Because heart-rate-variability (HRV) analysis requires continuous interbeat-interval information (Shaffer and Ginsberg, 2017), no HRV indices were calculated. The data were also not used to infer specific autonomic mechanisms.

### 2.5 Acoustic analysis

Acoustic analysis was conducted in Praat version 6.4.38 (Boersma and Weenink, 2025). For the purpose of constructing the present acoustic-stability score, lower values of pitch standard deviation (Pitch SD), jitter, and shimmer, together with higher values of harmonics-to-noise ratio (HNR), were treated as indicating greater voice consistency.

All four acoustic parameters were z-standardized across all observations. The directions of the component measures were then aligned so that higher standardized values consistently represented greater voice consistency within the composite. A composite acoustic-stability score was subsequently calculated as:


$$\begin{array}{l} Composite=-z\left(PitchSD\right)-z\left(Jitter\right)-z\left(Shimmer\right)+z\left(HNR\right) \end{array}$$

Higher Composite values indicated greater acoustic stability. After directional alignment, the four standardized measures were combined using equal unit weights. This operational aggregation should not be interpreted as evidence that the four measures made equal empirical contributions to acoustic stability; their relative contributions were not examined because the analysis focused on transition-level changes in the composite score.

To obtain a bounded representation of acoustic stability, the Composite values were further standardized across all observations as follows:


$$\begin{array}{l} Composite_{z}=\frac{Composite-\mu_{comp}}{\sigma_{comp}} \end{array}$$

where μ_*comp*_ and σ_*comp*_ denote the overall mean and standard deviation of the Composite values, respectively.

The standardized Composite values were then transformed using the cumulative distribution function (CDF) of the standard normal distribution:


$$\begin{array}{l} Composite_{cdf}=\text{Φ}\left(Composite_{z}\right) \end{array}$$

where Φ denotes the cumulative distribution function of the standard normal distribution.

For reporting and subsequent analyses, *Composite*_*cdf*_ was expressed as a CDF-scaled acoustic-stability score ranging from 0 to 100:


$$\begin{array}{l} A=100\times Composite_{cdf} \end{array}$$

The resulting variable *A* was used as the acoustic-stability score in all subsequent register-transition comparisons and physiological–acoustic coupling analyses. Higher values of *A* indicated greater acoustic stability on the CDF-scaled acoustic-stability score. The construction of *A* is summarized in Figure 1, and the resulting values are reported in Supplementary Table S1.

In the present study, *A* served as a bounded operational measure of acoustic stability during the controlled sustained-vowel tasks. It combined directionally aligned information from Pitch SD, jitter, shimmer, and HNR to capture relative differences across register segments within the same participant and arousal block. This focus was consistent with the study's aim of examining transition-level acoustic change rather than evaluating overall vocal quality or artistic performance. The analysis therefore focused on within-participant, within-block source-to-target changes in *A*, rather than on absolute rankings between participants. Transition-level acoustic change (Δ*A*) is defined in Section 2.6.2.

### 2.6 Transition-level analysis

#### 2.6.1 Signed maximal short-timescale HR slope within each register segment

Short-timescale heart-rate (HR) slopes were computed between adjacent HR observations spaced at 10-s intervals. For each time step t, the HR change was defined as:


$$\begin{array}{l} s_{t}=HR_{t+1}-HR_{t}\;\left(bpm\;per\;10\;s\right) \end{array}$$

For each register's singing segment, the maximal short-timescale HR slope (*s*_max_) was defined as the HR change with the largest absolute magnitude:


$$\begin{array}{l} s_{\max}=s_{t^{†}},\text{ where }|s_{t^{†}}|=\underset{t}{\max}|s_{t}| \end{array}$$

The signed value of *s*_max_ was retained. A positive value indicates a dominant short-timescale HR increase, whereas a negative value indicates a dominant short-timescale HR decrease within that register segment.

If an isolated missing value prevented a 10-s adjacent computation, the slope was computed over the nearest valid pair and normalized to a 10-s equivalent (e.g., a 20-s HR difference was divided by 2 to yield bpm per 10 s). The specific pair used was documented during annotation.

#### 2.6.2 Register-transition comparisons

Analyses were conducted at the level of register-transition events within each arousal block. For each participant, two transitions were defined per block:

- Low register → Middle register
- Middle register → High register

Each event linked a source register segment with the subsequent target segment, preserving the correspondence among participant, arousal block, transition type, and the two register segments.

For each register segment, short-timescale HR fluctuation was indexed by the signed maximal short-timescale HR slope (*s*_max_), as defined in Section 2.6.1. Retaining the signed value allowed source-to-target differences to reflect changes in sign, magnitude, or both.

For each register transition (source → target), two quantities were calculated:

1. HR-slope trajectory shift, defined as the difference in signed maximal short-timescale HR slope between the target and source registers:
$$\begin{array}{l} \Delta s=s_{\max}^{target}-s_{\max}^{source} \end{array}$$
2. Acoustic stability change, defined as the difference in the CDF-scaled acoustic-stability score between the target and source registers:

$$\begin{array}{l} \Delta A=A^{target}-A^{source} \end{array}$$

Here, “HR-slope trajectory shift” is used descriptively to denote the numerical source-to-target difference in signed maximal short-timescale HR slope. Δ*s* was therefore not interpreted as a simple increase or decrease in mean HR, while Δ*A* represented the corresponding source-to-target change in acoustic stability.

Both quantities were calculated within the same participant and arousal block, thereby preserving the correspondence among participant, arousal block, and transition type while reducing the influence of between-participant baseline differences. The analysis did not assume that the relationship between Δ*s* and Δ*A* was uniform across participants or task contexts, and their association was not used to infer a specific autonomic mechanism or causal direction.

#### 2.6.3 Coupling classification

Each transition was encoded using a pairwise coupling code:

HR component:

- → if Δ*s* > 0, indicating a rightward HR-slope trajectory shift;
- ← if Δ*s* < 0, indicating a leftward HR-slope trajectory shift;
- = if Δ*s* = 0, indicating no net HR-slope trajectory shift.

Acoustic component:

- + if Δ*A* > 0, indicating that acoustic stability increased from source to target;
− if Δ*A* < 0, indicating that acoustic stability decreased from source to target.

This yields four possible coupling patterns:


$$\begin{array}{l} \left(\to ,+\right),\;\left(\to ,-\right),\;\left(\leftarrow ,+\right),\;\left(\leftarrow ,-\right) \end{array}$$

For higher-order descriptive grouping, ( →, +) and (←, −) were classified as aligned, whereas ( →, −) and (←, +) were classified as opposed. Events with Δ*s* = 0 were reported separately as tie events and were not included in the aligned or opposed totals.

These codes summarized the paired directions of transition-level changes in signed maximal short-timescale HR slope and acoustic stability rather than absolute HR or acoustic values. They were descriptive and did not imply causality, physiological valence, or that aligned coupling was preferable to opposed coupling. Event-level coupling codes for all participants and arousal blocks are reported in Table 2.

**Table 2:** Event-level coupling distribution across arousal conditions.

ID	Low arousal	Moderate arousal	High arousal
1	[( →,+), ( →,−)]	[(←,+), ( →,−)]	[(←,+), ( →,−)]
2	[(←,+), ( →,−)]	[(←,+), ( →,−)]	[(←,+), ( →,−)]
3	[(←,+), (←,+)]	[(←,+), ( →,+)]	[( →,+), (←,+)]
4	[(←,+), ( →,−)]	[(←,+), ( →,−)]	[(←,+), ( →,+)]
5	[(←,+), ( →,−)]	[(←,+), ( →,−)]	[( →,+), (←,−)]
6	[( →,+), (←,+)]	[( →,+), ( →,+)]	[(←,+), ( →,+)]
7	[( →,+), (←,+)]	[( →,+), (←,−)]	[( →,+), (←,−)]
8	[(←,+), ( →,−)]	[( →,+), (←,−)]	[(←,+), ( →,+)]
9	[( →,+), ( →,+)]	[(←,−), ( →,+)]	[( →,−), ( →,+)]
10	[( →,+), (←,+)]	[( →,+), ( →,+)]	[( →,+), (←,−)]
11	[( →,+), ( →,+)]	[(←,+), ( →,+)]	[(←,+), ( →,+)]
12	[( →,+), ( →,+)]	[(←,+), ( →,+)]	[( →,+), (←,+)]
13	[( →,−), (←,+)]	[(←,−), ( →,+)]	[(←,+), ( →,+)]
14	[( →,+), ( →,−)]	[( →,+), ( →,−)]	[( →,+), ( →,−)]
15	[( →,+), (←,+)]	[( →,+), ( →,+)]	[( →,+), ( →,+)]
16	[(←,+), ( →,+)]	[( →,+), ( →,+)]	[( →,+), ( →,−)]
17	[(←,+), ( →,+)]	[(←,+), ( →,+)]	[( →,+), ( →,+)]
18	[(←,−), ( →,−)]	[( →,−), ( →,−)]	[( →,−), (←,+)]
19	[( →,+), (←,+)]	[(←,+), ( →,+)]	[( →,+), (←,−)]
20	[( →,+), ( →,−)]	[(←,+), ( →,−)]	[( →,+), ( →,−)]
21	[(←,+), ( →,+)]	[(←,+), (←,+)]	[(←,+), (←,+)]
22	[( →,+), ( →,+)]	[(←,+), ( →,+)]	[(=,+), (←,+)]
23	[( →,−), ( →,+)]	[(←,−), ( →,−)]	[(←,−), (←,+)]
24	[( →,+), ( →,−)]	[(←,+), ( →,−)]	[(←,+), ( →,−)]
25	[(←,+), ( →,+)]	[(←,+), ( →,+)]	[(←,+), (←,+)]

ID, participant identifier; HR, heart rate. Within each cell, the first pair refers to the Low→ Middle transition and the second pair to the Middle→ High transition. LA, low arousal; MA, moderate arousal; HA, high arousal. For the HR component, → indicates Δ*s* > 0, ← indicates Δ*s* < 0, and = indicates Δ*s* = 0, where Δ*s* is the signed maximal short-timescale HR slope in the target register minus that in the source register. For the acoustic component, + indicates Δ*A* > 0 and − indicates Δ*A* < 0, where Δ*A* is the acoustic-stability score in the target register minus that in the source register. Aligned patterns are ( →,+) and (←,−); opposed patterns are ( →,−) and (←,+). Events with Δ*s* = 0 were classified separately as tie events and were not included in the aligned or opposed categories. The single tie event was coded (=,+).

For individual-profile classification, two criteria had to be satisfied simultaneously. First, at least four of the participant's six transition events had to belong to the same higher-order coupling category, either aligned or opposed. Second, at least two of the three arousal blocks had to show the same block-level tendency.

For the second criterion, a block was classified as aligned when aligned events outnumbered opposed events within that block, as opposed when opposed events outnumbered aligned events, and as mixed when the counts were equal. Tie events were excluded when determining the block-level tendency.

A participant was classified as aligned-dominant only when at least four of the six transition events were aligned and at least two arousal blocks showed an aligned block-level tendency. A participant was classified as opposed-dominant only when at least four events were opposed and at least two blocks showed an opposed block-level tendency. Participants who did not satisfy both conditions for either category were classified as mixed. Tie events were not counted toward either dominant category.

### 2.7 Statistical analysis

Transition-level analyses were conducted at the register-transition event level, with repeated observations nested within participants. Coupling frequencies were summarized as aligned, opposed, or tie events. For the group-level comparison between aligned and opposed coupling, the aligned-minus-opposed proportion difference and its 95% confidence interval were estimated using participant-cluster bootstrap resampling. Participants, rather than individual transition events, were resampled as intact clusters to preserve the dependence among observations contributed by the same participant. Tie events were reported separately and excluded from the aligned-vs.-opposed proportion calculation.

The continuous association between transition-level change in signed maximal short-timescale HR slope (Δ*s*) and change in acoustic stability (Δ*A*) was examined using Pearson's correlation. Spearman's rank correlation was additionally calculated as a sensitivity analysis to assess whether the direction and approximate strength of the association depended on the use of a linear correlation coefficient. Because each participant contributed multiple transition events, the 95% confidence interval for Pearson's correlation was estimated using participant-cluster bootstrap resampling.

A second sensitivity analysis was conducted at the participant-by-transition level. For each participant and transition type, event-level Δ*s* and Δ*A* values were averaged across the LA, MA, and HA blocks, yielding 50 participant-by-transition observations. Pearson's correlation, Spearman's rank correlation, and the participant-cluster bootstrap confidence interval were then calculated for the aggregated data. The resulting relationship is presented in Supplementary Figure S1.

The arousal-manipulation check was conducted using participant-level mean singing HR across the LA, MA, and HA blocks. These values were evaluated using repeated-measures analysis of variance. Sphericity was assessed using Mauchly's test; when the assumption was violated, Greenhouse–Geisser-corrected degrees of freedom were reported together with partial η^2^. A Friedman test with Kendall's W was conducted as a non-parametric sensitivity analysis. Individual coupling-profile proportions were accompanied by Wilson 95% confidence intervals.

All participant-cluster bootstrap 95% confidence intervals were based on 10,000 resamples. Hypothesis tests were two-sided, with α = 0.05. Interpretation emphasized effect estimates and 95% confidence intervals where applicable.

Data management and statistical analyses were performed in Python 3.12.13 using NumPy 2.5.1 (Harris et al., 2020), pandas 3.0.3, SciPy 1.18.0 (Virtanen et al., 2020), and statsmodels 0.14.6 (Seabold and Perktold, 2010).

## 3 Results

### 3.1 Event counts and arousal-manipulation check

Analyses were conducted at the register-transition event level within each arousal block. Each participant contributed two transition events in each block: one Low→ Middle transition and one Middle→ High transition. This yielded 50 events per block and 150 events in total across the LA, MA, and HA blocks.

Each event was classified according to the directions of change in signed maximal short-timescale HR slope (Δ*s*) and acoustic stability (Δ*A*), and was subsequently categorized as aligned, opposed, or tie according to the rules described in Section 2.6.3. Events with Δ*s* = 0 were reported separately as tie events and were not included in the aligned or opposed totals. Event-level coupling codes are reported in Table 2.

Participant-level mean singing HR was 104.62 bpm (SD = 15.03) in the LA block, 102.22 bpm (SD = 12.43) in the MA block, and 105.99 bpm (SD = 12.76) in the HA block. Mauchly's test indicated a violation of sphericity, *W* = 0.374, χ^2^(2) = 21.74, *p* < 0.001; therefore, Greenhouse–Geisser-corrected results are reported. The repeated-measures ANOVA showed a significant block effect, *F*(1.23, 29.51) = 5.66, *p* = 0.018, partial η^2^ = 0.191. The Friedman sensitivity analysis also indicated differences among blocks, χ^2^(2) = 12.56, *p* = 0.002, Kendall's *W* = 0.251. Both analyses indicated an overall difference in mean singing HR among the three blocks. However, the block means did not increase progressively from LA to MA to HA, and the manipulation should not be interpreted as producing a uniform physiological dose across participants. Participant-level values and individual trajectories are shown in Supplementary Table S2 and Supplementary Figure S2.

### 3.2 Directional distributions of HR-slope trajectory shifts and acoustic-stability changes

Across the 150 register-transition events, rightward HR-slope trajectory shifts were more frequent than leftward shifts in each arousal block. In LA, 33 events showed rightward shifts and 17 showed leftward shifts; in MA, the corresponding counts were 30 and 20. In HA, 27 events showed rightward shifts, 22 showed leftward shifts, and 1 event was a tie. Across blocks, rightward shifts occurred in 54 of 75 Middle→ High events and 36 of 75 Low→ Middle events. These counts describe the direction of change in signed maximal short-timescale HR slope and do not indicate the magnitude of that change.

Positive changes in acoustic stability occurred in 38 of 50 LA events, 35 of 50 MA events, and 37 of 50 HA events. Across blocks, positive Δ*A* occurred in 65 of 75 Low→ Middle events and 45 of 75 Middle→ High events. Because transition-level changes in the acoustic-stability score were calculated within participants, these counts describe the direction of source-to-target change rather than differences in absolute stability between participants.

### 3.3 Event-level coupling and continuous association

Coupling was summarized by the joint directions of Δ*s* and Δ*A*. In the LA block, 23 events were aligned and 27 were opposed; in MA, 25 were aligned and 25 were opposed; and in HA, 24 were aligned, 25 were opposed, and 1 was a tie.

Across all 150 transition events, 72 were classified as aligned, 77 as opposed, and 1 as a tie. Excluding the tie event, the aligned proportion was 0.483 and the opposed proportion was 0.517. The aligned-minus-opposed proportion difference was −0.034, with a participant-cluster bootstrap 95% confidence interval of [−0.230, 0.160]. Because this interval included zero, the data did not support a single dominant coupling direction at the group level.

The HA block contained one tie event, coded (=,+), for which Δ*s* = 0 and Δ*A* > 0. It was reported separately and did not alter the qualitative conclusion of approximate balance.

**Figure 2:**
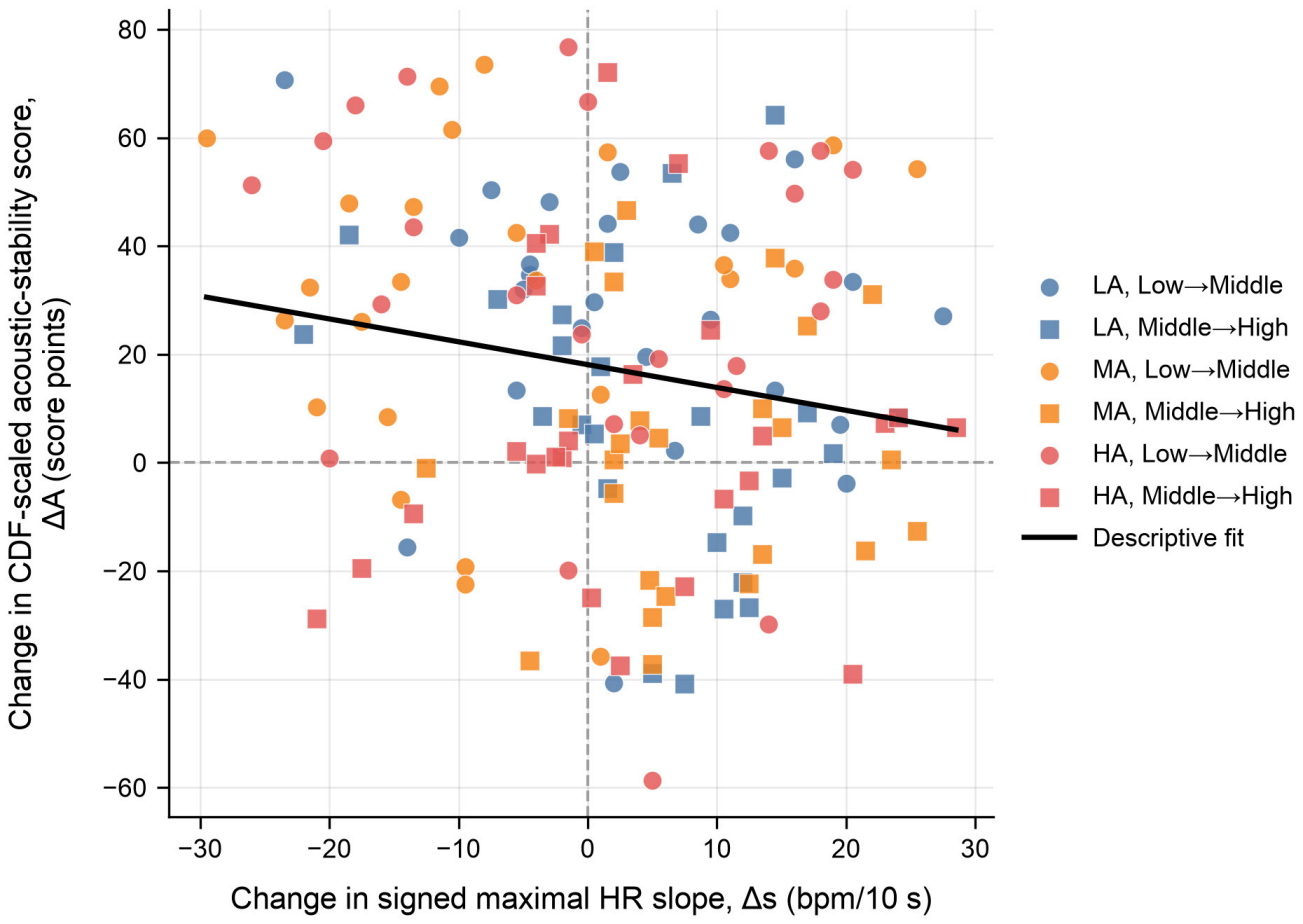
*Continuous event-level relationship between change in signed maximal short-timescale HR slope and change in the CDF-scaled acoustic-stability score*. Each point represents one register-transition event contributed by one participant in one arousal block, yielding 150 events in total. The x-axis shows Δ*s*, calculated as the signed maximal short-timescale HR slope in the target register minus that in the source register. The y-axis shows Δ*A*, calculated as the CDF-scaled acoustic-stability score in the target register minus that in the source register. Colors indicate arousal blocks, and marker shapes indicate transition type. Dashed lines mark zero change on each axis. The fitted black line provides a descriptive visual summary of the continuous association and is not interpreted as evidence of causality. LA, low arousal; MA, moderate arousal; HA, high arousal; HR, heart rate; CDF, cumulative distribution function.

To complement the categorical coupling codes, the continuous event-level relationship between Δ*s* and Δ*A* was examined across all 150 register-transition events (Figure 2). Pearson's correlation indicated a weak negative association, *r* = −0.180, with a participant-cluster bootstrap 95% confidence interval of [−0.317, −0.040]. Spearman's rank correlation showed a similar weak negative association, ρ = −0.177. The bootstrap interval remained below zero when participants, rather than individual events, were resampled as intact clusters. Nevertheless, the association was weak and descriptive and did not imply that changes in HR slope caused changes in acoustic stability.

As a sensitivity analysis, Δ*s* and Δ*A* were averaged across the three arousal blocks for each participant and transition type, yielding 50 participant-by-transition observations. The resulting association is shown in Supplementary Figure S1. The aggregated association remained weakly negative, Pearson's *r* = −0.283, with a participant-cluster bootstrap 95% confidence interval of [−0.493, −0.043]; Spearman's ρ = −0.309.

### 3.4 Individual coupling profiles

Using the dominance criterion described in Section 2.6.3, 7 of 25 participants were classified as opposed-dominant [28.0%; Wilson 95% CI (14.3%, 47.6%)], 4 as aligned-dominant [16.0%; Wilson 95% CI (6.4%, 34.7%)], and 14 as mixed [56.0%; Wilson 95% CI (37.1%, 73.3%)]. More than half of the participants were therefore classified as mixed, whereas the remaining participants met either the aligned-dominant or opposed-dominant criterion. This distribution indicates heterogeneity in individual coupling profiles rather than convergence on one common physiological–acoustic pattern.

## 4 Discussion

### 4.1 Short-timescale HR fluctuation as a descriptive regulatory marker in singing

The present study examined how transition-level changes in signed maximal short-timescale HR slope co-varied with changes in acoustic stability during singing. The continuous event-level analysis showed a weak negative association between Δ*s* and Δ*A*, while aligned and opposed categorical coupling patterns occurred at comparable frequencies. Together, these findings indicate a weak but observable relationship between HR-slope change and acoustic-stability change, without supporting one dominant group-level coupling direction.

The HR-slope measure should be interpreted as a descriptive marker of short-timescale regulatory change rather than as a direct measure of effort, stress, autonomic mechanism, or vocal quality. Register transitions can involve coordinated adjustments in vocal production and respiratory–laryngeal behavior (Titze, 2000; Traser et al., 2022), but the present data do not establish that changes in HR slope caused changes in acoustic stability. Instead, the results show that the two forms of transition-level change co-varied across task events.

This event-based approach complements block-level mean HR summaries by retaining information about how short-timescale HR trajectories changed across successive register segments. At the same time, the observed associations were modest and individual coupling profiles were heterogeneous. Short-timescale HR fluctuation is therefore best understood here as one component of a person- and task-sensitive regulatory pattern, rather than as a uniform physiological response shared across singers.

### 4.2 No single dominant group-level coupling direction

Aligned and opposed coupling occurred at comparable frequencies across the three arousal blocks. At the group level, the aligned-minus-opposed proportion difference was small, and its participant-cluster bootstrap confidence interval included zero. These results do not support one dominant coupling direction at the group level.

The continuous event-level analysis nevertheless showed a weak negative association between Δ*s* and Δ*A*. Thus, the absence of a dominant categorical direction should not be interpreted as an absence of structure. Rather, the continuous and categorical analyses describe complementary aspects of the same transition-level relationship.

The findings therefore suggest that the observed physiological–acoustic relationship is structured but non-uniform, rather than converging on one common HR-slope trajectory configuration.

### 4.3 Register-specific patterns and individual coupling profiles

The descriptive distributions differed between the two register-transition types. Middle→ High transitions showed a greater prevalence of rightward HR-slope trajectory shifts, whereas increases in acoustic stability were more frequent during Low→ Middle transitions.

These transition-specific differences should be interpreted in relation to the task-defined register contexts rather than pitch height alone. Because pitch served only as a within-participant task anchor, the present findings do not determine which specific vocal-coordination features accounted for the observed differences between transition types.

At the individual level, coupling profiles were heterogeneous. The predominance of mixed profiles indicates that the group-level pattern did not arise from a uniform participant-level response. At the same time, the aligned- and opposed-dominant groups show that some singers met the study's directional-consistency criterion in different ways. This distribution supports a person-sensitive interpretation of physiological–acoustic coupling rather than a single group-level response pattern.

### 4.4 Regulation as a trajectory rather than a target

The present findings support a trajectory-based rather than target-based interpretation of short-timescale physiological regulation during singing. In this framework, regulation is not defined by reaching a particular HR value or by displaying one preferred direction of HR change. Instead, it is described by how the signed maximal short-timescale HR slope changes across successive register segments.

This distinction is important because a rightward or leftward shift on the signed HR-slope axis can arise from different combinations of direction and magnitude. For example, a movement from a more negative to a less negative slope represents a rightward trajectory shift even when both register segments remain on the negative side of the axis. The arrow therefore summarizes the direction of transition-level change in signed maximal short-timescale HR slope rather than the magnitude of that change or a simple increase or decrease in mean HR.

The approximately balanced aligned and opposed coupling frequencies, together with the weak continuous association between Δ*s* and Δ*A*, indicate that the observed relationship was not concentrated in one HR-slope trajectory configuration. Changes in acoustic stability were observed across multiple transition-level configurations. This interpretation is consistent with the observed individual heterogeneity and avoids assigning inherent physiological value to either rightward or leftward HR-slope trajectory shifts.

### 4.5 Interpretive boundaries and limitations

Several limitations define the scope of the present findings. First, the sample comprised 25 trained undergraduate singers aged 18–21 years, included an unequal distribution of voice types, and was limited to students specializing in Western classical singing or Chinese national singing. The broadly comparable training background and health status of the participants supported controlled within-participant comparisons but did not eliminate individual differences in vocal technique, register-control difficulty, or physiological response. Register-control difficulty was not measured independently and could therefore not be examined as a participant-level explanatory factor. Although each participant completed the same Low, Middle, and High task structure, these task-defined settings should not be interpreted as technically equivalent across voice categories. For example, a task-defined Low or High setting may involve a different technical emphasis for a baritone than for a tenor or soprano. Because the voice-type groups were small and unevenly represented, such between-category differences could not be examined systematically in the present study. The findings should not be generalized directly to untrained singers, professional singers, broader age groups, individuals with voice disorders, or singers working in other vocal traditions and styles, such as musical theater or popular singing.

Second, the LA, MA, and HA blocks represented structured task contexts rather than identical physiological doses. Although participant-level mean singing HR differed across blocks, individual patterns were not uniform and the block means did not increase progressively from LA to MA to HA. The arousal manipulation should therefore be interpreted as creating distinguishable physiological contexts rather than standardized arousal levels shared equally by all participants. In addition, the blocks were administered in a fixed LA → MA → HA order, so block-related differences cannot be fully separated from possible order, fatigue, or carryover effects.

Third, HR was measured using a first-generation Apple Watch Ultra and analyzed as interval-averaged estimates at 10-s time steps. Wrist-PPG measurements provide useful estimates of average HR, but their agreement with ECG reference measurements can vary with movement, activity context, and measurement window (Alfonso et al., 2022; Charlton et al., 2022; Lambe et al., 2026). The present analysis was therefore restricted to short-timescale changes in the exported 10-s HR series and did not estimate beat-to-beat variability or specific autonomic mechanisms. Future studies using ECG or validated beat-to-beat recordings could provide finer temporal resolution and independent assessment of the observed HR-slope patterns.

Fourth, the acoustic-stability score combined four selected measures using equal unit weights. In the context of the controlled sustained-vowel tasks, the score provided an operational representation of relative voice consistency across register segments within the same participant and arousal block. Its interpretation should remain tied to this experimental purpose rather than being extended to an overall evaluation of vocal quality or artistic performance. The score should therefore not be generalized unconditionally beyond the present task context or interpreted as a context-free standard that supersedes perceptual, stylistic, or artistic judgment.

Finally, the observed associations between Δ*s* and Δ*A* were modest and correlational. They indicate that short-timescale HR-slope changes and acoustic-stability changes varied together across register-transition events, but they do not establish that one caused the other. The findings therefore support structured but non-uniform physiological–acoustic coupling rather than a fixed causal mechanism or a single optimal regulatory direction.

### 4.6 Future directions

The present findings motivate future work using denser repeated-measures designs and larger, more diverse samples. Repeated productions across arousal blocks, register transitions, token lengths, sessions, and stages of training would make it possible to estimate the stability, variability, and context dependence of each singer's HR–acoustic trajectory. Extending recruitment beyond trained undergraduate singers would also allow the framework to be examined across broader age groups, training levels, voice types, vocal styles, and levels of register-control experience.

Future studies should also examine a wider range of vocal and aesthetic tasks. Sustained vowels and predefined register transitions provide a controlled experimental setting, but they do not represent the full range of demands involved in singing. Melodic passages, texted singing, different languages, stylistic conventions, musical character, expressive direction, timbral goals, and contrasting aesthetic criteria may each alter the relationship between physiological regulation and acoustic output. Such designs would help determine which coupling patterns are specific to the present task and which generalize across performance contexts.

The framework could further be extended by examining how physiological and acoustic measurements relate to expert judgments, behavioral evidence, and singers' reported experiences. The present results suggest that transition-level acoustic changes cannot be interpreted through one fixed mapping from HR-slope direction alone. Computational models used for measurement, pattern detection, or feedback should therefore preserve the distinction between measurable signals and their contextual interpretation. Within this broader agenda, the present study should be viewed as an initial methodological step toward person-sensitive physiological–acoustic monitoring in vocal training, rather than as a ready-to-use diagnostic or pedagogical tool.

Such work could support person-specific, human-in-the-loop approaches to vocal assessment, in which computational methods assist with evidence organization and pattern detection while human experts retain responsibility for contextual interpretation and final evaluative judgment (Li, 2026). The aim would not be to prescribe one optimal physiological state or universal scoring rule, but to examine which combinations of physiological, acoustic, experiential, and expert evidence are informative for particular singers, tasks, and aesthetic aims.

Higher-resolution physiological recording would remain valuable for determining whether the observed 10-s HR-slope patterns correspond to finer temporal features of respiratory–cardiac coordination. Greater temporal precision alone, however, would not resolve the interpretive challenge. Future research should therefore combine improved measurement with broader participant groups, more ecologically varied vocal tasks, and transparent models of human–machine evaluation.

## 5 Conclusion

This study examined short-timescale HR fluctuation and acoustic stability during register-transition events in trained singers. By comparing source-to-target changes in signed maximal short-timescale HR slope (Δ*s*) with changes in the acoustic-stability score (Δ*A*), the analysis extended beyond block-level mean HR summaries and categorical coupling codes alone.

Aligned and opposed coupling occurred at comparable frequencies at the group level, while the continuous event-level analysis showed a weak negative association between Δ*s* and Δ*A*. Individual coupling profiles were heterogeneous, with more than half of the participants classified as mixed and the remaining participants meeting either the aligned- or opposed-dominant criterion. Together, these results indicate a modest but observable physiological–acoustic structure without supporting one dominant group-level coupling direction.

Short-timescale HR fluctuation should therefore be interpreted as a descriptive marker of regulatory change rather than as a direct measure of effort, vocal quality, autonomic mechanism, or causal influence. More broadly, the findings support a trajectory-based and person-sensitive view of physiological–acoustic regulation in singing. The event-based framework provides a basis for future work using broader vocal tasks, more diverse samples, higher-resolution physiological measurements, and multimodal approaches that integrate measurable signals with expert and contextual interpretation.

## Data Availability

The original contributions presented in the study are included in the article/Supplementary material, further inquiries can be directed to the corresponding author.
