# Supplementary material for: Short-timescale heart-rate fluctuation and acoustic stability show structured coupling at register transitions during singing
Source: Front Psychol. 2026 Aug 13;17:1783731. doi: 10.3389/fpsyg.2026.1783731 (PMC13518162; doi:10.3389/fpsyg.2026.1783731)
Supplement: Supplementary file 1 [file Supplementary_file_1.docx]

**Supplementary Material**

**Supplementary Figure S1. Participant-by-transition aggregated relationship between change in signed maximal short-timescale HR slope and change in the CDF-scaled acoustic-stability score.**

Each point represents one participant and one register-transition type, with event-level $\Delta s$ and $\Delta A$ averaged across the LA, MA, and HA blocks, yielding 50 participant-by-transition observations. Each participant therefore contributed one Low→Middle observation and one Middle→High observation. Marker shapes distinguish the two transition types. The fitted black line provides a descriptive visual summary only. LA, low arousal; MA, moderate arousal; HA, high arousal.

**Supplementary Figure S2. Participant-level mean singing HR across the LA, MA, and HA blocks.**

Light-gray points and connecting lines show participant-level mean singing HR across the three arousal blocks. Black points and lines indicate block means, with error bars representing t-based 95% confidence intervals for the block means. The plotted values are participant-level singing-block means and do not include transition-level observations. LA, low arousal; MA, moderate arousal; HA, high arousal; HR, heart rate.
